# Multi-criterion analysis of the effect of physico-chemical microbiological agents on *Legionella* detection in hotel water distribution systems in Crete

**DOI:** 10.3389/fcimb.2023.1214717

**Published:** 2023-12-22

**Authors:** Dimosthenis Chochlakis, Vassilios Sandalakis, Apostolos Ntoukakis, Maria-Olga Daskalaki, Thomas Loppinet, Niki Thalassinaki, Rena Makridaki, Christos Panoulis, Anna Psaroulaki

**Affiliations:** ^1^Regional Laboratory of Public Health of Crete, School of Medicine, University of Crete, Heraklion, Greece; ^2^Laboratory of Clinical Microbiology and Microbial Pathogenesis, School of Medicine, University of Crete, Heraklion, Greece

**Keywords:** Legionella, water, model, physico-chemical agents, microbiological agents

## Abstract

**Introduction:**

Water distribution systems in hotels have been related to outbreaks caused by *Legionella* spp. Certain measures, including disinfection by chlorination, maintaining increased temperatures are usually undertaken to prevent *Legionella* outbreaks. However, these preventive strategies are not always effective, since there are several factors (e.g., synergistic interactions with other microbes, physico-chemical factors, biofilm formation, availability of nutrients) that promote survival and proliferation of the pathogen in water pipes., Accordingly, there is a need of a holistic approach in development of preventive models for *Legionella* outbreaks associated with water distribution systems.

**Methods:**

Water samples were collected from hotel water systems and were tested for the presence of *Legionella*, *E. coli*, total coliforms, total mesophilic count and *Pseudomonas*. In each sample, temperature and chlorine were also tested. Other epidemiological factors were additionally recorded including number of rooms, stars, proximity of sampling point to the boiler, etc. Data were processed by generalized linear analysis, and modeling based on logistic regression analysis to identify independent predictive factors associated with the presence of *Legionella* in hotel water systems.

**Results:**

According to the generalized linear model, temperature affected (p<0.05) the presence of *Legionella* regardless of the species or the water supply (hot or cold). Additionally, opportunistic (*P. aeruginosa*) or non-opportunistic (*E. coli*, coliforms) pathogens were significantly associated (p<0.05) with the presence of all *Legionella* species. Temperature also exhibited a positive effect to all pathogens tested except for *Pseudomonas* according to the linear model. Multivariate analysis showed that *Pseudomonas*, total coliforms, HPC and temperature had a statistically significant effect on the presence of *Legionella*. Based on a binomial model, cold water had a positive effect on *Legionella*. Type of sampling and proximity of the sample to the boiler seemed to pose different effect on *Legionella* depending on the cfu/L. The number of hotel stars and rooms did not appear to have any effect in all tested models.

**Discussion:**

Collectively, these results indicate the need for development of individualized water safety plans tailored by the presence of other microbiological agents, and unique physico-chemical factors, which could facilitate the survival of *Legionella*.in hotel water systems.

## Introduction

*Legionella* is a genus of Gram-Negative bacteria, which contains over 50 different species and is a part of the gamma proteobacteria class (Schwake et al., 2015). Legionella species are the causative agents of either Pontiac Fever, a flu like non-fatal disease, or Legionnaires’ Disease (LD), an atypical form of pneumonia with much more severe symptoms which can be fatal. *Legionella pneumophila* is the commonest cause of LD, accounting for almost 85% of cases (Papagianeli et al., 2021; Kermani et al., 2022). The most common transmission route of LD is through inhaling of *Legionella* contaminated aerosols, resulting in subsequent pulmonary infection (Kermani et al., 2022) (Volker et al., 2016). As the notification rate for Legionellosis has risen to 1.8/100.000 which is the highest ever recorded in the EU, it is critical to prevent the *Legionella* contamination of artificial water systems, which pose the biggest threat for human infection (Papagianeli et al., 2021; Kermani et al., 2022).

*Legionella* species’ natural reservoirs are freshwater and wet soil, where the bacteria survive mostly as intracellular parasites of free-living protozoans, especially amoeba species such as *Acanthamoeba* (Rogers et al., 1994; Borella et al., 2005). However, *Legionella’s* most important reservoirs are man-made water environments including piped drinking water, cooling towers, fountains and humidifiers, with hot water systems being colonized more often than cold water and hotel hot water systems being related to Travel Associated LD (Borella et al., 2005; Volker et al., 2016; Rasheduzzaman et al., 2020). In artificial aquatic environments, *Legionella* colonization has been associated with the presence of biofilms (Borella et al., 2005; Bargellini et al., 2011), which protect *Legionella* from disinfectants and contain the protozoa in which *Legionella* replicates. In the biofilm environment, the abundance of nutrients allows for *Legionella* to grow extracellularly, also (Schwake et al., 2015).

*Legionella* in hotel water systems may pose a great risk especially to immunocompromised and debilitated individuals. Furthermore, hotels often have a high occupancy rate and a constant turnover of guests. This means that if *Legionella* is present in the water system, many people may be exposed to it over time, increasing the risk of an outbreak. Of crucial importance is the complexity of plumbing systems in the hotels, including numerous water fixtures, pipes, and tanks. *Legionella* bacteria thrive in warm water, and these systems can provide ideal conditions for their growth and spread of the pathogen. Moreover, water in some parts of the system may stagnate, creating pockets of stagnant water where *Legionella* can multiply. Since *Legionella* bacteria can become aerosolized in showers, hot tubs, water spectacles, etc., guests may get infected by inhalation of contaminated water droplets. Finally, there are always legal and reputational concerns since if a guest contracts Legionnaires’ disease while staying at a hotel, it can lead to legal liability and significant damage to the hotel’s reputation. Lawsuits, investigations, and negative media coverage can result from outbreaks associated with hotels.

To mitigate the risk of Legionella in hotel water systems, many jurisdictions have implemented regulations and guidelines for hotel operators to follow, including regular monitoring, water treatment, and maintenance practices to ensure the safety of their guests. Proper water management and *Legionella* prevention measures are essential to protect both guest health and the hotel’s reputation. To ensure that the above-mentioned criteria are met, several factors need to be considered when trying to minimize the risk of water systems from *Legionella*.

Various studies have associated several physio-chemical factors with the survival of *Legionella* and the formation of biofilms in pipeline surfaces with the colonization of *Legionella* in an attempt to create mathematical models that predict the risk of the bacterium’ s growth and human exposure, models which would help develop control strategies to prevent legionellosis (Yunana et al., 2021). Despite the fact that most “traditional” entero-pathogens, enter artificial water systems in the rare case of sewage system malfunction or greater water age (Falkinham et al., 2015; De Giglio et al., 2021), *Legionella* are considered opportunistic environmental pathogens, a distinct category of pathogens, similarly to *Mycobacterium* spp., and *Pseudomonas aeruginosa*. If certain conditions are met, these pathogens are able to survive disinfection procedures and grow in the oligotrophic environment of engineered water, even in low concentrations of oxygen, posing a health threat especially for the immunocompromised people (Lu et al., 2017; Zhang et al., 2021; Zhang et al., 2021a). The most important risk factors identified so far include high water temperature (Rasheduzzaman et al., 2020), water stagnation, pipeline material and roughness, structure age, nutrient concentration, such as Zn and Mn, disinfectant concentration, pipeline length and corrosion and Heterotrophic Platelet Count (HPC) (Bargellini et al., 2011; Volker et al., 2016).

The aim of the current study was to implement different mathematical approaches to try and obtain conclusive results on the effect of several physico-chemical and microbiological factors on the presence of *Legionella* in water distribution systems.

## Materials and methods

### Hotel selection

The study took place during the period 2018-2022. The hotels that participated in the study were distributed at the four prefectures (Lassithi, Heraklion, Rethymno, Chania) of the island of Crete, southern Greece. Hotels were chosen either based on routine surveillance or following the notification of a human case of *Legionella* infection in a tourist (Travel-Associated *Legionella* Disease) from ECDC and the national ministry of health. The hotels were distributed at the four prefectures of the island based on the number of hotels accommodated in each prefecture and on the corresponding population. An effort was also made to distribute the hotels according to city- or non-city ones and on seasonal or annual operation.

For each hotel, the sorting according to stars was recorded; the number of rooms, the date of sampling, whether the sampling was the first one or not, and the mode of operation (annual or seasonal) were also recorded.

The permanent population of the region where each hotel was hosted was retrieved from the database of the Hellenic Statistical Authority (https://www.statistics.gr/en/home/).

For each hotel, the geographical coordinates were recorded to map the results at a later stage and try to come to certain conclusions on the presence of *Legionella* that could be due to the locality of the hotel, the local public water distribution system, the local climate, etc. These data are not shown for reasons that have to do with the confidentiality of results.

### Sampling

The number of samples tested from each hotel were calculated based on the complexity of the water distribution system, the number of boilers and the size of the establishment. In general, one water sample (tap water) was collected from the following sites: inlet of each hotel water system, right after the main water supply tank, water returning or leaving the boiler, and the closest and the farthest, from the boiler, rooms. The samples from the rooms (room showers) were collected following a one-minute flush for the cold and a two-minute flushing for the hot water. Direct sampling, collecting samples right after opening the showers, was not carried out to avoid any misleading results that could be due to a dirty end.

Water samples were collected in sterile plastic bottles (1 liter each) that contained 20mg of sodium thiosulfate to neutralize the residual-free chlorine. All water samples were stored at 4°C and processed within 24h of collection.

### *Legionella* isolation-detection-identification

Isolation of *Legionella* from water samples was performed by culture according to the International Standard method ISO 11731 (1998), the ISO 11731-2 (2004) and the ISO 11731:2017. Based on our experience as a reference laboratory for water quality assessment for the island of Crete, the application of the latest ISO only, may lead to false negative or false positive results.

Briefly, water samples were concentrated by filtration through a 0.22μm pore diameter sterilized polyethersulfone membrane (Sartorius, Germany). After filtration, bacteria collected on the membranes were re-suspended in 10 ml 1/40 Ringer’s solution in 50ml falcon tubes and vortexed for two minutes. Two hundred microliters were spread on BCYE (buffered charcoal yeast extract with α-ketoglutarate, L-cysteine and ferric pyrophosphate), BCY (buffered charcoal yeast extract without L-cysteine) and GVPC (agar supplemented with vancomycin, polymyxin B, cycloheximide and glycine) (Oxoid, ThermoFisher Scientific) petri dishes, directly after filtration, after incubation at 50°C for 30 minutes and after addition of acid buffer. The inoculated plates were incubated for 10 days at 36 ± 1°C in 5% CO_2_ with increased humidity and were checked at two, five, seven and ten days. Based on our protocol, the cut-off for considering a positive result was set at 50 cfu/L.

Suspected colonies were further processed by MALDI Biotyper (Microflex LT MALDI-TOF mass spectrometer, Bruker Daltonics, Germany) for identification of *Legionella* species. Agglutination testing (Prolex-Lab Diagnostics, Waltham, USA), was also applied to discriminate *L. pneumophila* serogroup 1 from serogroups 2-14 (and each serogroup in separate) and *Legionella* species.

### Isolation-detection-identification of other microorganisms

Apart from *Legionella*, water samples were also tested for the presence of *E. coli*, coliforms, *Pseudomonas* and Total Mesophilic bacteria.

The ISO 9308 (2014) was followed for the isolation, detection and enumeration of *E. coli* and total coliforms. Briefly, 100 ml of water sample were filtered through a 0.45μm pore diameter sterilized polycarbonate membrane (Pall Corporation, Michigan, USA). The membrane was placed onto a Chromogenic Coliform Agar (CCA) and was incubated at 36 ± 2°C for 24 hours. *Escherichia coli* were identified based on color whereas coliforms were confirmed by oxidase testing. *Escherichia coli* isolates were further confirmed by MALDI-TOF-MS analysis. The results were expressed as number of *E. coli* and/or total coliforms colonies/100ml.

The ISO 16266 (2009) was followed for the isolation, detection, and enumeration of *Pseudomonas*. Briefly, 100ml of water samples were concentrated by filtration through a 0.22μm pore diameter sterilized polycarbonate membrane (Pall Corporation, Michigan, USA). The membrane was placed onto a *Pseudomonas* agar base/CN-agar and was incubated at 36 ± 2°C for 22 ± 2 44 ± 4 hours. All plates were checked under UV light (UV λ=360 ± 20nm). Typical *P. aeruginosa* isolates and isolates positive to the Nessler’s reaction were further confirmed by MALDI-TOF-MS analysis. The results were expressed as number of *Pseudomonas* colonies/100ml.

The ISO 6222 (1998) was followed for the isolation and enumeration of Total Mesophilic Bacteria. Briefly, 1ml of each water sample was spread onto a Yeast Extract Agar plate, in duplicate. The first plate was incubated at 36 ± 2°C for 44 ± 4 hours while the other at 22 ± 2°C for 68 ± 4 hours. The number of colonies was recorded, and the results were expressed as number of colonies/ml.

### MALDI-TOF mass spectrometry

MALDI Biotyper (Microflex LT MALDI-TOF mass spectrometer) (Bruker Daltonics, Leipzig, Germany) equipped with a microSCOUT ion source, was used for identification of individual *Legionella* colonies against the microbial database (v3.1.2.0). Spectra were recorded using the flexControl software with the default parameters set by the manufacturer for optimization (Bruker Daltonics, Leipzig, Germany). For each spectrum, 240 laser shots were collected and analyzed (6 × 40 laser shots from different positions of the target spot. All identifications were evaluated according to the manufacturer scoring scheme. *Escherichia coli* ATCC 8739 was used as an internal control.

### Physico-chemical testing

At the time of sampling free residual chlorine and temperature were recorded. The Lovibond MD 100 chlorine, LR/HR, ClO_2_ instrument was used for the recording of free residual chloride. The Testo 206 (Testo, Germany) was used for the recording of temperature. Both instruments were calibrated since our laboratory is accredited for all the above-mentioned methodologies.

### Statistical analysis

Quantitative data were extracted for certain biological and environmental parameters focusing on the impact of temperature, HPC and number of rooms as the environmental factors and of Chlorine, *E. coli*, Coliforms, *P. aeruginosa* and *Pseudomonas* species as the biomarkers in the search of any relationship with the presence of *Legionella*. An attempt was made to build a multivariate regression model that would predict the presence and variability in the numbers of *Legionella* bacteria in water distribution systems.

All the analyses were performed using statistics packages in R version 3.6.1 environment to build Binomial and Poissons/Quasi-Poissons models. Pscl was used to develop Zero-inflated and Hurdle models and Ade4 for the development of descriptive models. OptimalCutpoints was used to test for different values as cut-off points. Dbscan and fpc were used for clustering of data.

The Benjamini-Hochberg Procedure (B-H) correction was applied to decrease the false discovery rate and to make sure that small p-values (less than 5%) do not happen by chance (false positives).

## Results

A total of 663 samples were tested over the four-year period of 2018-2022. The samples were collected from 38 different hotels from the four prefectures of the island of Crete distributed as follows: seven (7) from Lassithi (eastern Crete), nine (9) from Heraklion (central Crete), four (4) from Rethymno (western Crete) and 18 from Chania (western Crete).

Of the samples tested, 107 (16%) were positive for *Legionella* (regardless the serogroup, or species), 12 (1.8%) were positive for *E. coli*, 102 (15.4%) were positive for total coliforms, 73 (11%) were positive for P. aeruginosa and 107 (16%) for *Pseudomonas* species.

Apart from *L. pneumophila* sg 1, five (5) other serogroups were also, detected in the water samples tested; these were serogroups 3, 6, 8, 9, and 10. The dominant of all serogroups including 1, was 3 (it was detected in 39 out of the 663 samples tested; 5.9%). In fact, during the last decade, serogroups 3, 6 and 8 have dominated the isolation rate in water samples tested in Crete. Serogroup 14 has almost disappeared while several species are not frequently isolated any more. A large metagenomic analysis is undertaken to figure out the reasons hiding behind that. As regards Legionella species, *L. anisa*, *L. londiniensis*, *L. taurinensis* and *L. erythra* were isolated with their rates ranging not more than one percentage of the total samples. These results along with the range of the cfu/L are shown in **Table 1**.

**Table 1:** Isolation rate of *L. pneumophila* and L. species.

Species/serogroup	No tested samples	Positive	% positive*	% positive^	cfu/L range
L. p. 1	**663**	35	5.3		50-8750
L. p. 2-14		92	13.9		50-5550
L. p. 3		39	5.9	42.4	550-5550
L. p. 6		27	4.1	29.3	350-4250
L. p. 8		23	3.5	25.0	200-3500
L. p. 9		2	0.3	2.2	50-250
L. p. 10		1	0.2	1.1	50
L. species		11	1.7		50-1050
*L. anisa*		4	0.6	36.4	50-150
*L. londiniensis*		3	0.5	27.3	50-350
*L. taurinensis*		9	0.3	18.2	300-850
*L. erythra*		11	0.3	18.2	550-1050

The range of colony forming units (cfu/L) for each species/serogroup is also shown. L. p. 1: *L. pneumophila* serogroup 1; L. p. 2-14: *L. pneumophila* serogroups 2-14. *: as per total number of samples tested. ^: as per intra-serogroup/species.

**Figure 1:**
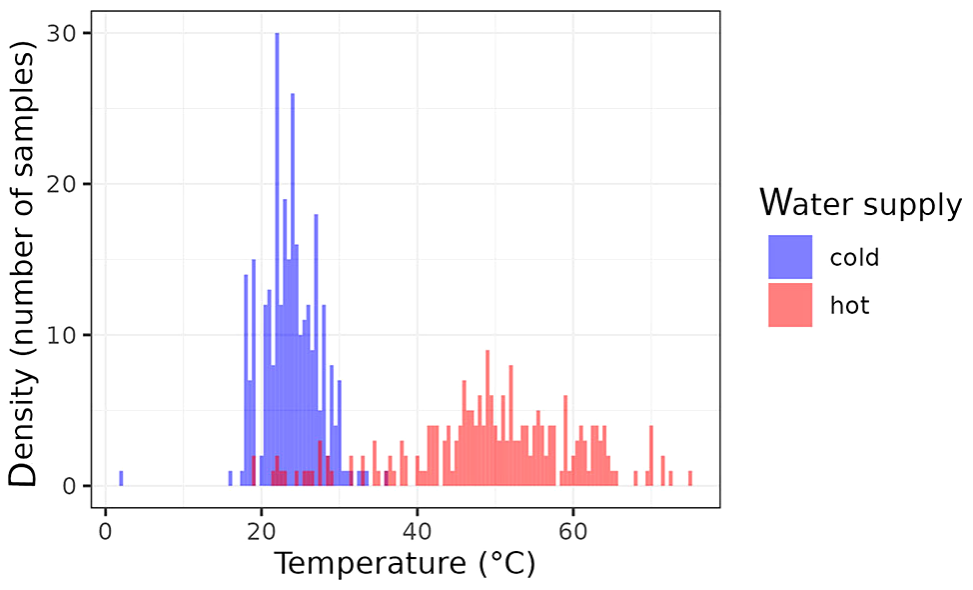
Distributions of the water temperatures of analyzed samples (red: hot water; blue: cold water).

The distribution of the water samples collected according to their temperature is shown at **Figure 1**. Some of the samples collected from hot water supplies were far away from being considered as hot ones since their temperatures were very low. On the other hand, very few cold-water samples fulfilled the criterion of less than 20°C.

### Cut-off points

The variability in the data could affect the robustness of the models that we intended to develop. To avoid misleading data points, yet without excluding the outliers that could be crucial, optimal cut-point analysis was used as an extension of ROC analysis, to make the transition from quantitative variables to qualitative.

Certain cut-off point values were selected for either negative or positive (yes/no) test results. For the purposes of our analysis, the cost-benefit (CB) criterion was selected to calculate the slope S of ROC curve as the weight of the relative costs that multiple different predictions may have on the positive/negative result.

The following formula was used for the analysis: (McNeill et al., 1975; Metz et al., 1975; Metz, 1978)


$$S=\left(\left(1-p\right)/p\right)*CR$$

**Table 2** presents the results of OptimalCut Analysis with no categorical variable as co-variate, and with hot and cold-water systems as categorical variable respectively.

**Table 2:** Cut-off points using ROC and OptimalCut: a) with no categorical variable as co-variate; b) at hot water systems; c) at cold water systems.

	Variable	Breaks
No categorical variable	*Legionella*	[0, 1000], (1000, 16000]
	HPC	[0, 20], (20, 1000]
	Free chlorine (mg/L)	[0, 0.2], (0.2, 8.4]
	No of rooms	(0, 100], (100, 200], (200, 563]
	Temperature	(0, 25], (25, 50], (50, 75]
	*E. coli*	[0], (0, 42]
	Coliforms	[0], (0, 300]
	*P. aeruginosa*	[0], (0, 350]
	*Pseudomonas* species	[0], (0, 250]
Hot water systems	No of Stars	[0, 4], (4, 6]
	No of rooms	[0, 132], (132, 564]
	Free chlorine (mg/L)	[0, 0.11], (0.11, 9.4]
	Temperature	[0, 47.4], (47.4, 76]
	*E. coli*	[0, 0], (0, 43]
	*Pseudomonas* species	[0, 1], (0, 251]
	Coliforms	[0, 1], (0, 301]
	*P. aeruginosa*	[0, 1], (0, 351]
	HPC	[0, 13], (13, 1000]
Cold water systems	No of rooms	[0, 134], (134, 564]
	Free chlorine (mg/L)	[0, 0.16], (0.16, 9.4]
	Temperature	[0, 24.3], (24.3, 76]
	*E. coli*	[0, 1], (1, 43]
	*Pseudomonas* species	[0, 1], (1, 251]
	Coliforms	[0, 1], (1, 301]
	*P. aeruginosa*	[0, 1], (1, 351]
	HPC	[0, 21], (21, 1000]

### Linear model

A generalized linear model was initially used to test for the effect of temperature on the presence of *Legionella*, of *L. pneumophila* and of *L. pneumophila* sg 1. A binomial model was adjusted considering a linear effect of temperature and considering that the effect of temperature will be different depending on the water supply (hot or cold). This model was applied to estimate the effect of temperature on the probability of presence of *Legionella*, of *L. pneumophila* and of *L. pneumophila* sg 1 and the probability of detecting Total *Legionella* over the legal limit as described in the national legislation (1000 cfu/L).

The results presented (**Table 3**) indicate significant differences in the effect of temperature based on the water system supply. Specifically, at the same temperature, the estimated log-odds were significantly higher in hot water systems compared to cold water systems, as evidenced by the models Leg Tot Nor, Leg Tot Pre, L. p. Tot Pre, and L. p. sg1 Pre (with estimates of 6.58; p: 0.0016, 5.23; p <0.001, 6.06; p <0.001, 8.44 p:0.0013). Moreover, increasing temperature exhibited distinct effects on cold and hot water systems. While an increase in temperature augments the probability of *Legionella* presence in cold water systems, a corresponding increase in temperature appeared to decrease the probability of *Legionella* presence in hot water systems. These findings highlight the critical role of temperature in *Legionella* colonization and can inform strategies to mitigate *Legionella* growth in water systems.

**Table 3:** Estimated log-odds and associated p-value based on a generalized linear model that estimated the effect of temperature on the odds for the presence of *Legionella* (all *Legionella* assigned as Leg Tot Pre), *L. pneumophila* (assigned as L. p. Tot Pre) *L. pneumophila* sg 1 (assigned as L. p. sg1 Pre), and for *Legionella* lying withing normal values (assigned as Leg Tot Nor) according to the national legislation (1000 cfu/L).

Variable	Leg Tot Nor		Leg Tot Pre		L. p. Tot Pre		L. p. sg1 Pre	
	estimate	*p* value	estimate	*p* value	estimate	*p* value	estimate	*p* value
(Intercept)	-6.29	<0.001	-3.61	<0.001	-4.65	0.0012	-8.60	<0.001
Temperature	0.14	0.0671	0.09	0.0379	0.10	0.0922	0.21	0.0196
Cold water	6.58	0.0016	5.23	<0.001	6.06	<0.001	8.44	0.0013
Hot water	-0.18	0.0161	-0.15	0.0011	-0.16	0.0068	-0.27	0.0040

A binomial model was adjusted considering a linear effect of temperature and considering that the effect of temperature will be different depending on the water supply (hot or cold).

A similar correlation analysis was carried out to test for the effect of each of the other pathogens detected (*E. coli*, Coliforms, *P. aeruginosa*, *Pseudomonas* sp). Except for *Pseudomonas* species, the other three pathogen species seem to have a positive correlation with *L. pneumophila* sg 1, *L. pneumophila* and *Legionella* in total (**Table 4**).

**Table 4:** Correlation between presence of *Legionella* (*L. pneumophila* serogroup 1, *L. pneumophila* sgs 1-15 designated as L. p. total and all *Legionella* including *pneumophila* and species) and other bacteria using hi-squared test.

Variable	L. p. sg1			L. p. total			*Legionella* total		
	chi2	*p* value	adj p	chi2	*p* value	adj p	chi2	*p* value	adj p
*E. coli*	13.11	0.0095	0.024	5.21	0.0440	0.0733	10.75	0.0025	0.0031
Coliforms	13.50	<0.001	0.005	35.79	<0.001	0.0025	43.21	<0.001	0.0025
*P. aeruginosa*	6.05	0.0270	0.045	12.61	0.0025	0.0062	14.07	<0.001	0.0025
*Pseudomonas* sp	0.08	0.8051	1.000	1.83	0.1614	0.2018	12.23	0.0015	0.0025

The Benjamini-Hochberg Procedure (B-H) correction was applied at p-values to decrease the false discovery rate. Adj p: adjusted p-value.

**Figure 2:**
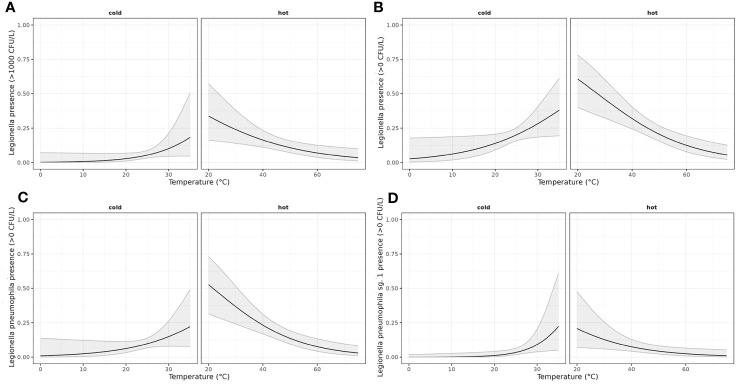
Generalized linear model estimating the effect of temperature on the odds of presence of LEGTOT, LPT, LP01 and LEGTOTNORM. A binomial model was adjusted considering a linear effect of temperature and allowing for a different effect of temperature depending on the water supply (hot or cold). **(A)** Estimated probability (with confidence interval of 95%) of LegTot being over norm depending on water supply (hot/cold) and temperature; **(B)** Estimated probability (with confidence interval of 95%) of LegTot being detected depending on water supply (hot/cold) and temperature; **(C)** Estimated probability (with confidence interval of 95%) of LPT being detected depending on water supply (hot/cold) and temperature; **(D)** Estimated probability (with confidence interval of 95%) of LP01 being detected depending on water supply (hot/cold) and temperature.

The results of the application of the linearized models are shown in **Figure 2**.

### Univariate and multivariate analysis

The extracted cut-off points were used as inputs in a univariate and multivariate binomial model. Each cut-off point of the biological or environmental parameters was used as the factor variable of exposure, while the cut-off point for *Legionella* was used as the response variable of the outcome. Given the exposure to the variable of interest, the odds of the outcome variable were computed, indicating whether the particular exposure could be considered as a risk factor of the observing outcome.

According to the OR estimates and the associated p-value (**Table 5**) following the univariate analysis for each of the characteristics against *L. pneumophila* sg 1, *L. pneumophila* and Total *Legionella* showed that the exposure to *Pseudomonas* and Coliforms as biological characteristics and to temperature and HPC as environmental parameters had a statistically significant (OR>1 and p-value <0.05) impact on the presence of *Legionella* regardless of the species.

**Table 5:** Univariate analysis for 10 variables against each of *L. pneumophila* sg 1 (L. p. sg 1), *L. pneumophila* and Total *Legionella* (*Legionella* species).

Variable	Parameter	Odds Ratio	p-value
*Pseudomonas* species	L. p. sg 1	3.42	0.09
	*L. pneumophila*	3.96	0.00
	*Legionella* species	4.58	0.00
*P. aeruginosa*	L. p. sg 1	0.75	1.00
	*L. pneumophila*	1.92	0.13
	*Legionella* species	1.72	0.16
Coliforms	L. p. sg 1	3.76	0.07
	*L. pneumophila*	4.40	0.00
	*Legionella* species	2.94	0.00
*E. coli*	L. p. sg 1	0.00	1.00
	*L. pneumophila*	0.98	1.00
	*Legionella* species	1.24	0.68
HPC	L. p. sg 1	6.65	0.01
	*L. pneumophila*	2.12	0.05
	*Legionella* species	2.70	0.00
Free chlorine (mg/L)	L. p. sg 1	0.46	0.44
	*L. pneumophila*	0.63	0.68
	*Legionella* species	0.16	0.00
Temperature	L. p. sg 1		0.09
	*L. pneumophila*		0.00
	*Legionella* species		0.00
12 month operation	L. p. sg 1	0.00	1.00
	*L. pneumophila*	1.25	0.59
	*Legionella* species	1.55	0.45
Hotel rooms	L. p. sg 1		0.25
	*L. pneumophila*		0.00
	*Legionella* species		0.02
Hotel stars	L. p. sg 1		0.08
	*L. pneumophila*		0.00
	*Legionella* species		0.00

It also seems that. among all the other exposure variables, the exposure to temperature (under 25°C for cold water and over 47°C for hot water) showed a high association with *Legionella*, reaching an OR up to 5.27 (**Table 6**).

**Table 6:** Univariate analysis for the effect of temperature against each of *L. pneumophila* sg 1 (L. p. sg 1) and Total *Legionella* (*Legionella* species). Cut-offs of 1000 and 50 cfu/L were chosen based on the recommendation of the national legislation and the LOD of the laboratory, respectively.

Parameter	Cut-off (cfu/L)	Odds Ratio	p-value
*Legionella* species	1000	2.66	0.00
	50	2.94	0.00
L. p. sg 1	1000	5.27	0.04
	50	4.83	0.00

**Figure 3:**
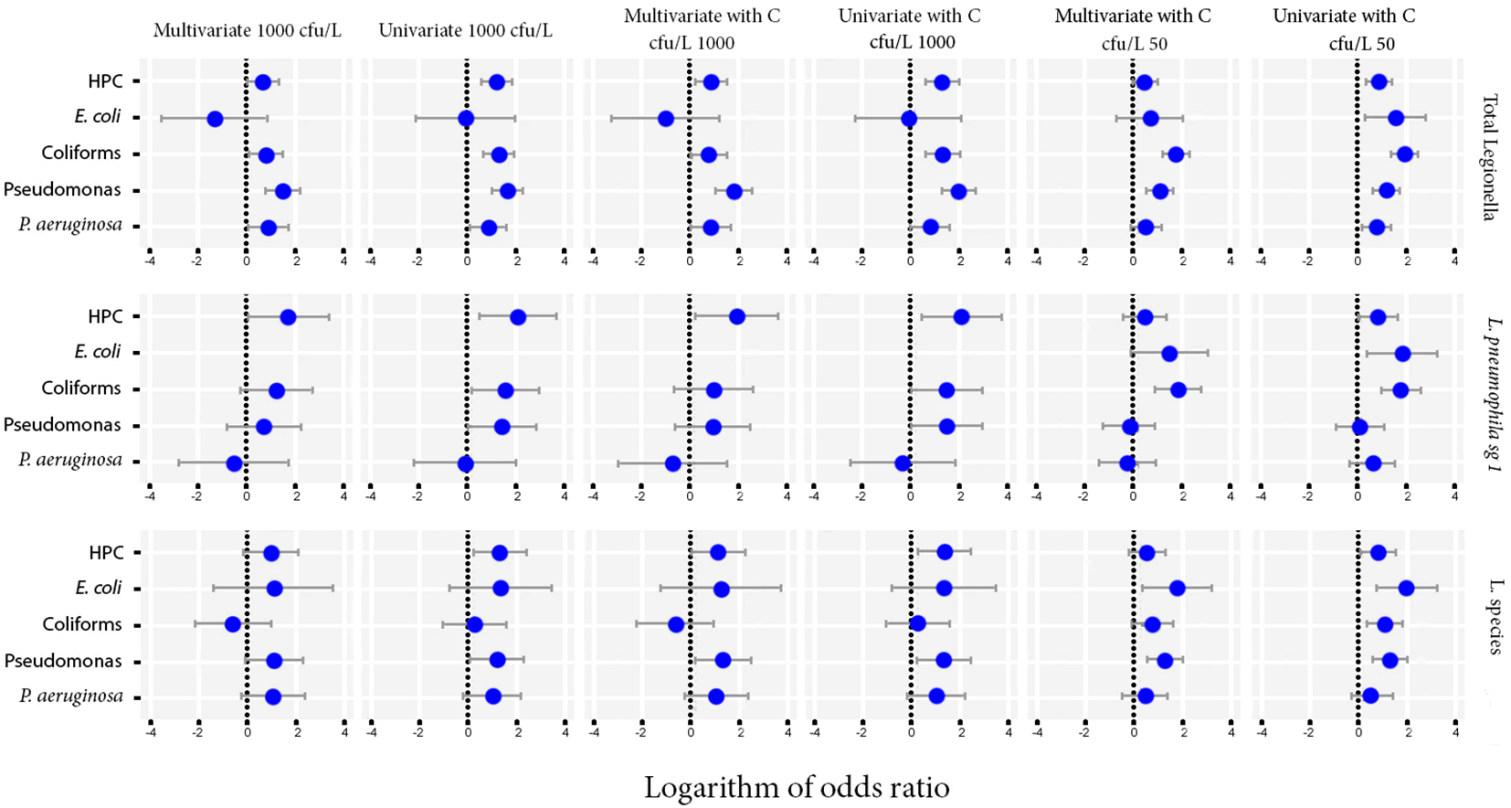
Odds ratios for the microbiological parameters of HPC, *E. coli*, Coliforms, *Pseudomonas* species and *P. aeruginosa* using univariate and multivariate models against Total *Legionella*, *L. pneumophila* sg 1 and *Legionella* species. Cut-offs of 1000 and 50 cfu/L were chosen based on the recommendation of the national legislation and the LOD of the laboratory, respectively.

Multivariate model was also performed as an extension of the odds ratio analysis in logistic regression, the results of which are shown as 95% CI (confidence intervals) at **Figure 3**.

**Figure 4:**
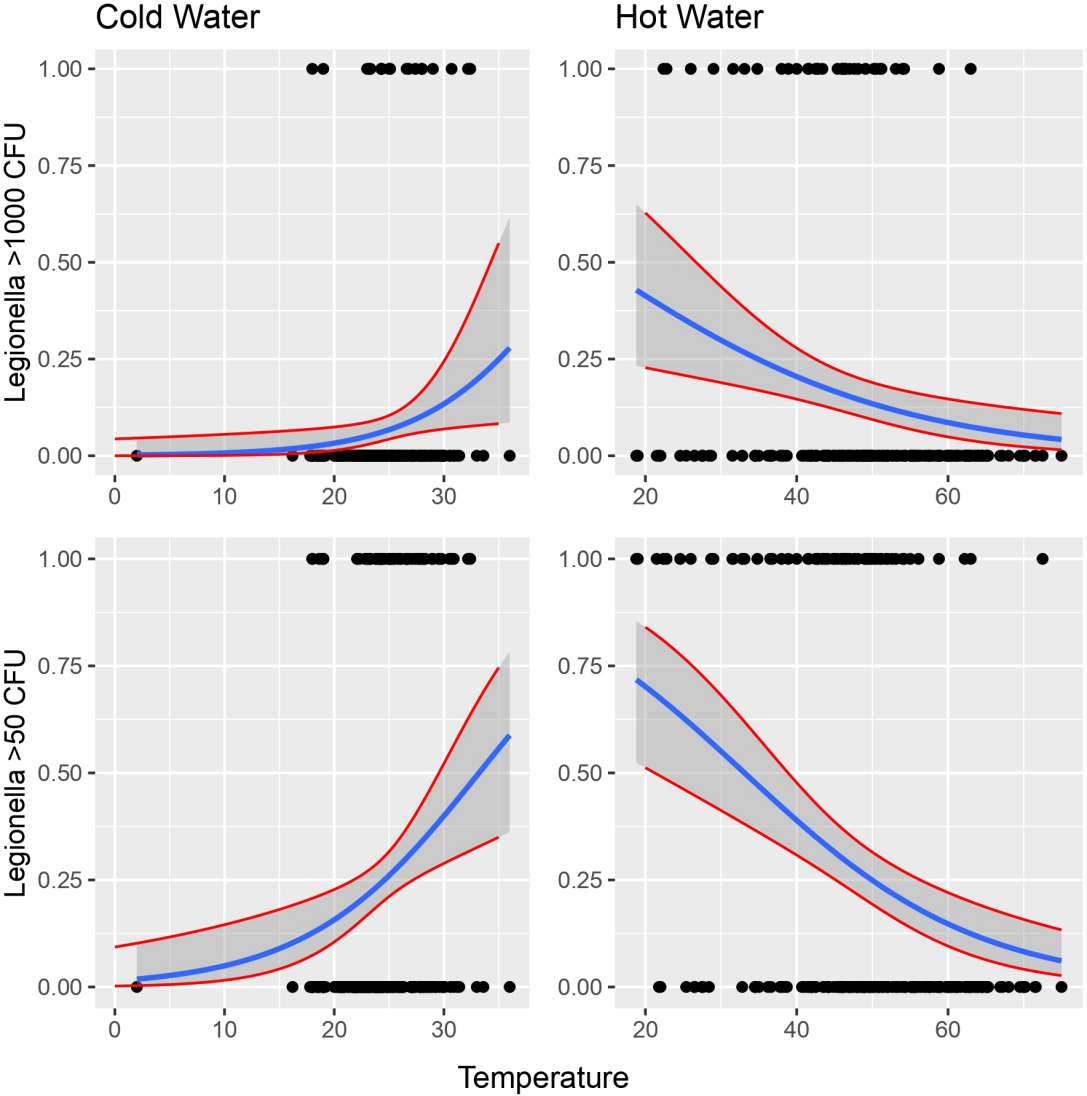
Possibility of detecting *Legionella* in the water according to temperature. Cut-offs of 1000 and 50 cfu/L were chosen based on the recommendation of the national legislation and the LOD of the laboratory, respectively.

The importance level was set at 0 to test for the simultaneous effect of HPC, *E. coli*, Coliforms, *Pseudomonas* species and *P. aeruginosa* under the covariate of temperature. The CI corresponding to those of HPC and *Pseudomonas* indicated a statistically significant association, while their small range depicts a higher precision of the OR metric (**Figure 4**).

The models hiding behind the above-mentioned analyses are given below.

For the multivariate model, the presence of *Legionella* together with the other five parameters could be described as:


$$▶logit\left(\mu_{i}\right)=\beta_{0}+\beta_{1}\times variable1_{i}+\beta_{2}\times variable2_{i}+\beta_{3}\times variable3_{i}\ldots$$

For the univariate model, only one parameter was taken into account each time:


$$▶logit\left(\mu_{i}\right)=\beta_{0}+\beta_{1}\times variable\Psi 1_{i},logit\left(\mu_{i}\right)=\beta_{0}+\beta_{2}\times variable2_{i},logit\left(\mu_{i}\right)=\beta_{0}+\beta_{3}\times variable3_{i}\;etc$$

If temperature is added into the model (multivariate model with temperature) then the odds of samples at the same level of temperature are compared:


$$▶logit\left(\mu_{i}\right)=\beta_{0}+\beta_{1}\times variable1_{i}+\beta_{2}\times variable2_{i}+\beta_{3}\times variable3_{i}\ldots +\beta_{temp}\times temp_{i}+\beta_{{\left(hot/cold\right)}_{i}}\times \left(hot/cold\right)+\beta_{interaction}\times {\left(hot/cold\right)}_{i}:temperature_{i}$$

When we used the univariate model for temperature, a single parameter was taken into account considering the differences due to temperature:


$$\begin{array}{l} ▶logit\left(\mu_{i}\right)=\beta_{0}+\beta_{1}\times variable1_{i}+\beta_{temp}\times temp_{i}+\beta_{{\left(hot/cold\right)}_{i}}\times \\ \left(hot/cold\right)+\beta_{interaction}\times {\left(hot/cold\right)}_{i}:temperature_{i}logit\left(\mu_{i}\right)=\beta_{0}+\\ \beta_{2}variable2_{i}+\beta_{temp}\times temp_{i}+\beta_{{\left(hot/cold\right)}_{i}}\times \left(hot/cold\right)+\\ \beta_{interaction}\times {\left(hot/cold\right)}_{i}:temperature_{i}\;etc\ldots \end{array}$$

Following all the above analyses, exposure to different water temperatures seemed to be the covariate with the greater association with *Legionella*. In terms of logistic regression, a binomial model was applied between *Legionella* as the response variables and water temperature as the binomial categorical independent variable with two values, hot water and cold water, as follows:


$$oddsi=exp\left(b0+b1*temperature\right)$$

Cold water seems to have positive linear relationship with *Legionella* while hot water seems to have negative linear relationship with the bacterium (**Figure 4**).

Using a binomial model, we supposed that under the logit scale, temperature has a linear power. In this case, the simple model would be as follows:


$$logit\left(\mu_{i}\right)=\beta_{0}+\beta_{1}\times temperature_{i}$$

which could also be written as:


$$odds_{i}=\text{exp}\left(\beta_{0}+\beta_{1}\times temperature_{i}\right)$$

making, however, the model more complicated since the parameter cold and hot water comes into play.

To deal with the excessive zero count data that may affect the robustness of our model, zero inflated and hurdle count regression analysis were performed using R pcsl package.

A ZI model assumed that zeros originated from two groups, “structural zeros” driven by the distribution itself and “sampling zeros” produced by the outcomes that were never reported. On the other side, a hurdle model assumed that all zeros are “structural”, as part of a Poisson or a truncated negative binomial distribution (Feng, 2021).

**Figure 5:**
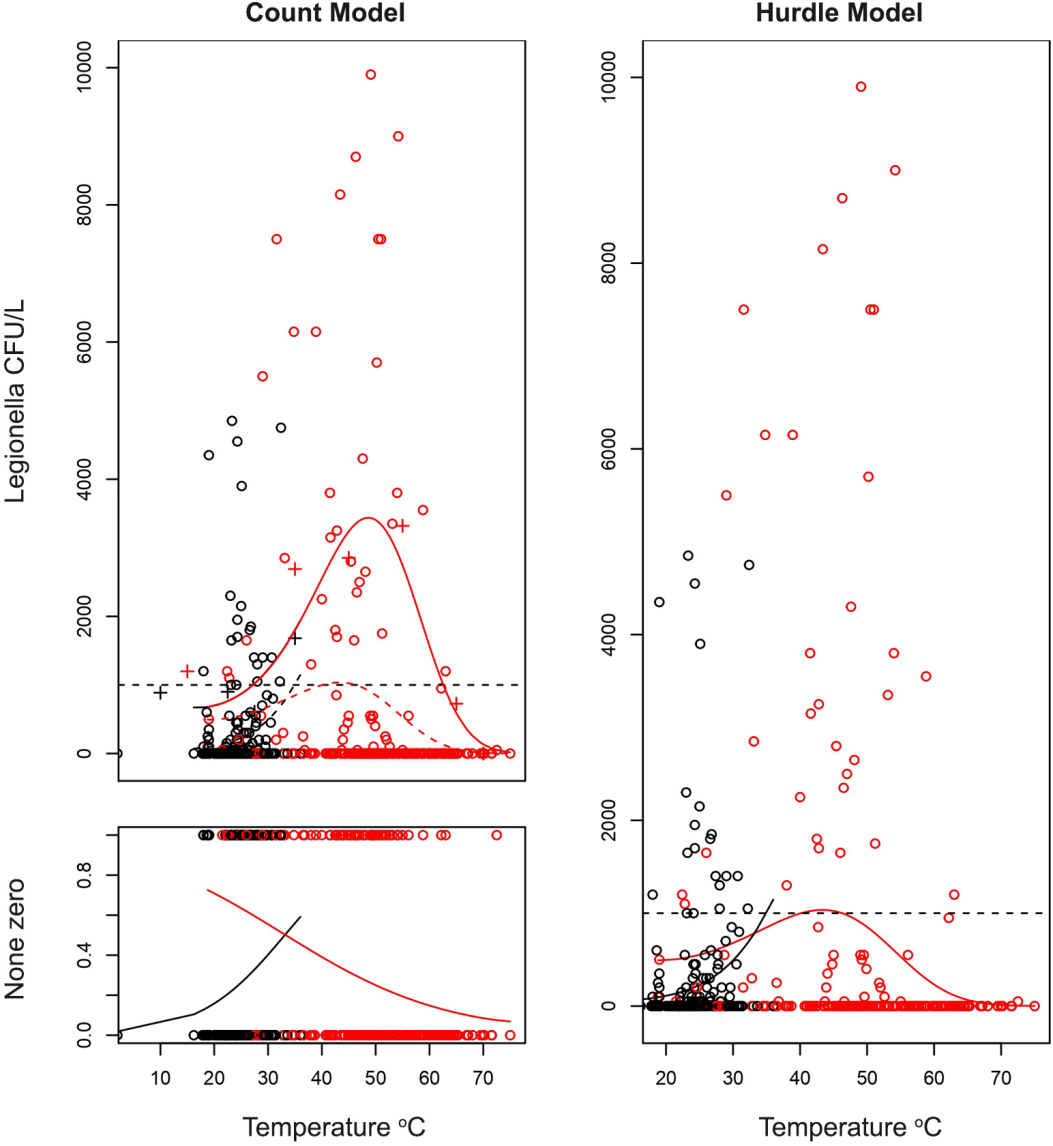
Estimations of quantitative effect of temperature on the count of *Legionella* CFU/L depending on the temperature of water. A hurdle model was adapted with the zero-prediction model and a polynomial model for the count data (coefficients on **Table 6**). On the first graph (top left) the fit of non-zero count data is presented. *Legionella* has an optimal temperature at which it best proliferates and can give very high CFU counts. However, as described in previous sections, the higher the temperature the more probable it is for the sample of being negative as described on the second figure (bottom left). Combining these two models gives the hurdle model on the right. Confidence intervals were not plotted, and the lines only represent the best fit. The red and black points are the samples with red corresponding to hot water and black to cold water samples.

Despite the different handling of zero occurrences between the two models applied (Count and Hurdle models), the results confirm the strong association between *Legionella* and water temperature as shown at the trend lines in **Figure 5**.

As regards the effect of chlorine, different models were applied considering chlorine either as a linear or a qualitive parameter. The different models were tested for each of Total *Legionella* or for *L. pneumophila* sg 1, using as cut-off values either the 1000 or the 50 cfu/L. Only Total *Legionella*, dis-regarding the cut-off value, revealed a statistically significant value and only when chlorine was treated as a qualitative parameter (**Table 7**).

**Table 7:** Analysis for the effect of chlorine against each of *L. pneumophila* sg 1 (L. p. sg 1) and Total *Legionella* (*Legionella* species).

Variable	Species	Cut-off	OR	p value
Linear	*Legionella*	1000	0.95	0.82
		50	1.06	0.65
	L. p. sg 1	1000	1.35	0.11
		50	1.46	0.00
Qualitative	*Legionella*	1000	0.16	0.00
		50	0.32	0.00
	L. p. sg 1	1000	0.46	0.32
		50	0.55	0.17
Qualitative	*Legionella*	1000	0.16	0.00
		50	0.30	0.00
	L. p. sg 1	1000	0.34	0.16
		50	0.58	0.21

Cut-offs of 1000 and 50 cfu/L were chosen based on the recommendation of the national legislation and the LOD of the laboratory, respectively.

**Figure 6:**
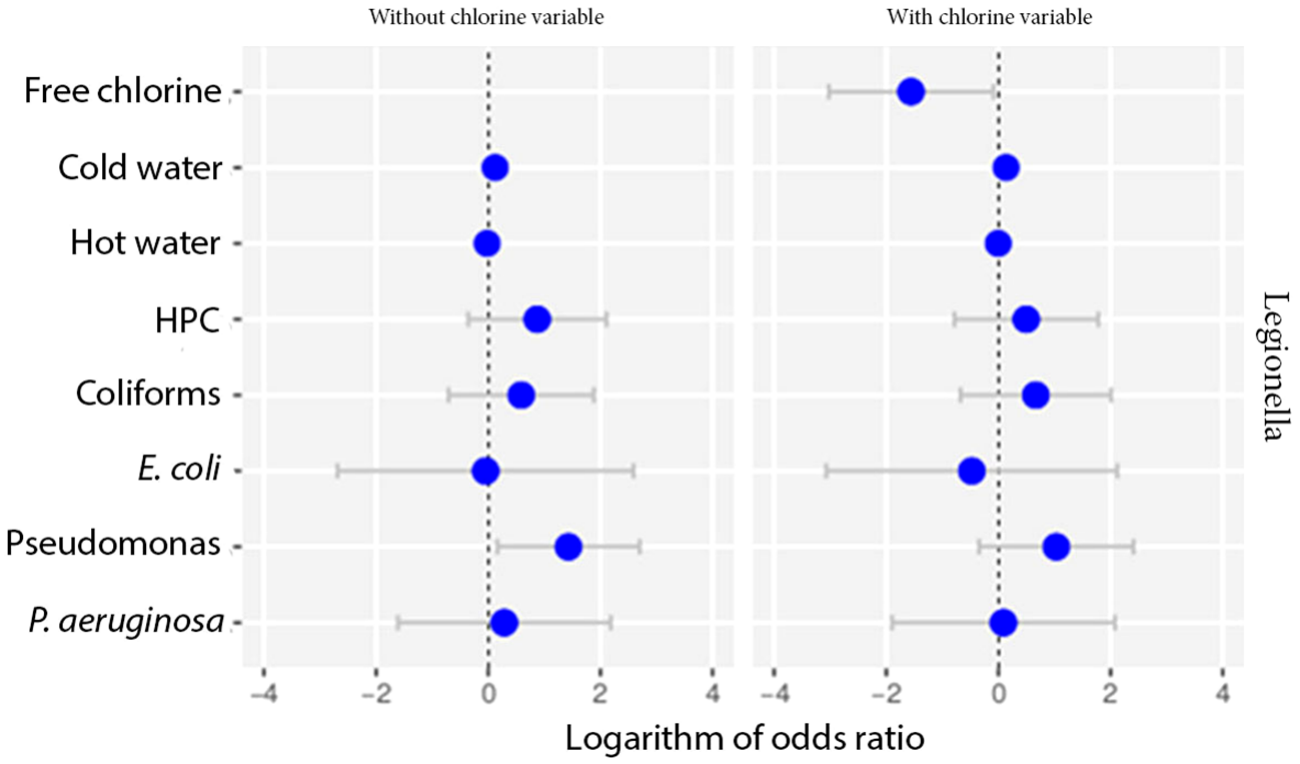
Odds ratio analysis testing the effect of chlorine against other parameters using multivariate models.

We also tested the data for potential differences among the samples in terms of the presence of other bacteria, that could be attributed to the presence or absence of chlorine. None of the parameters did not seem to produce any statistically significant result (**Figure 6**).

Further processing of the data by multivariate analysis with mixed quantitative variables revealed a strong correlation with temperature. *Pseudomonas aeruginosa* seems to prefer lower temperatures than *Legionella* for its survival and multiplication (data not shown).

The qualitative data of HPC, *Pseudomonas*, *E. coli*, *Legionella* against the physico-chemical parameters tested were further processed using Principal component analysis (PCA); the corresponding results are shown in a two-dimension scale. Cold water seems to positively affect *Pseudomonas*, whereas hot water exerts a negative effect at all pathogens tested, when chlorine and temperature are not considered as variables. Free chlorine seems to exert a similar effect on all pathogens. On the other hand, temperature acts in a different way based on whether the water is described as hot or cold (data not shown).

Apart from the microbiological and physico-chemical agents, the potential effect of other epidemiological factors (type of sample, number of stars and rooms of the hotel, number of hotels in the area tested) was also tested.

**Figure 7:**
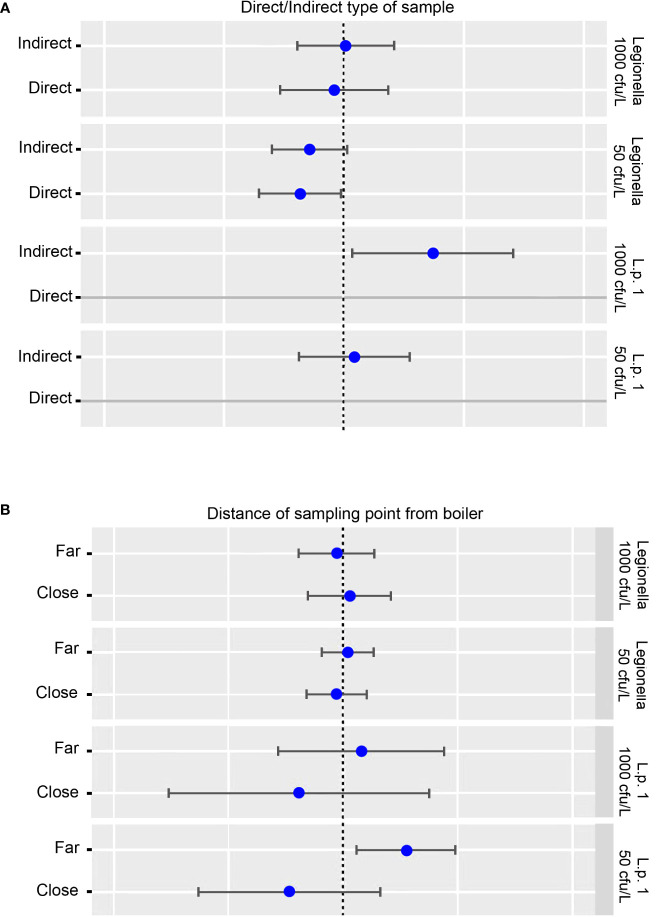
**(A)** Potential effect of direct and indirect sampling on *L. pneumophila* sg 1 (L. p. sg 1) and Total *Legionella* (*Legionella* species) presence. Cut-offs of 1000 and 50 cfu/L were chosen based on the recommendation of the national legislation and the LOD of the laboratory, respectively. **(B)** Potential effect of proximity of the sampling point to the boiler on *L. pneumophila* sg 1 (L. p. sg 1) and Total *Legionella* (*Legionella* species) presence. Cut-offs of 1000 and 50 cfu/L were chosen based on the recommendation of the national legislation and the LOD of the laboratory, respectively.

When testing for the effect of direct or indirect sampling, no relationship was calculated at a cut-off of 1000 cfu/L for *Legionella* and of 50 cfu/L for *L. pneumophila* sg 1. A negative effect was calculated at a cut-off of 50 cfu/L for *Legionella* and a positive effect at a cut-off of 1000 cfu/L (**Figure 7A**).

As regards the effect of proximity of the sample to the boiler, no effect was calculated when testing for *Legionella* regardless of the cut-off used. On the other hand, a positive effect was calculated for *L. pneumophila* sg 1 when testing the samples that were collected from sites far from the boiler, and a negative effect for samples close to the boiler. In both circumstances, the cut-off point did not seem to play any role (**Figure 7B**).

**Figure 8:**
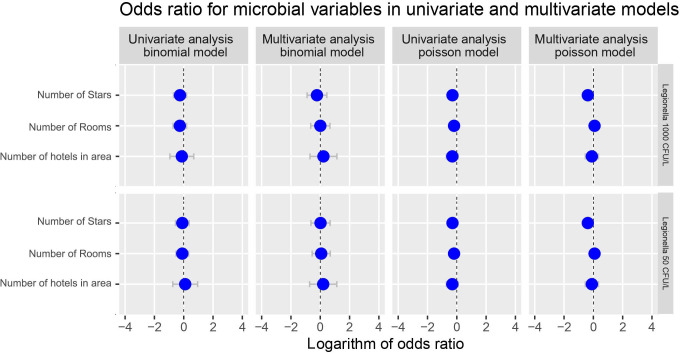
Potential effect of number of Stars and number of rooms of the hotel and the number of hotels situated at the area of sampling on Total *Legionella* (*Legionella* species) presence. Cut-offs of 1000 and 50 cfu/L were chosen based on the recommendation of the national legislation and the LOD of the laboratory, respectively.

The odds ratio analysis for the presence of *Legionella* compared to the number of stars and rooms of the hotel tested and of the number of hotels situated at the region of sampling did not seem to reveal and statistically significant effect irrespective of the model (binomial or Poisson) or the analysis (univariate or multivariate) chosen **Figure 8**).

On the contrary, when focusing strictly on the number of hotels tested in the area from which the samples were collected, a statistically significant p-value was calculated regardless the type of analysis followed (uni- or multi-variate) (**Table 8**).

**Table 8:** Odds ratio for Poisson model considering different aspects of the areas from which the samples were collected.

Coefficient Analysis	OR		p-value	
	Univariate	Multivariate	Univariate	Multivariate
No of hotels in the region tested	0.77	1.06	0.03	0.76
No of hotels sampled in the region tested	0.91	0.90	0.00	0.03

## Discussion

In this study we evaluated the effect of several factors that may influence the presence of *Legionella* in water distribution systems. We focused on temperature, since this is considered the most crucial one, chlorine, the presence of other pathogenic (*E. coli*, coliforms, *Pseudomonas*, *P. aeruginosa*) and non-pathogenic microorganisms (HPC), the type of operation of the hotel (annual or seasonal), other epidemiological factors (number of rooms, number of stars), and the type of sample collected (direct or following a two-minute flush). We also applied a holistic approach by implementing different mathematical analyses and models, to evaluate the potential significance and cross talk between the major risk factors.

We also performed individual analyses of the results on the presence of different *Legionella* spp., into separate for *L. pneumophila* sg 1, *L. pneumophila* (including all serogroups) and total *Legionella* (including any species or serogroup isolated). Moreover, we made our calculations based on both the LOD of our laboratory (50 cfu/L) and on the upper acceptable limit according to our National Legislation for *Legionella* (1000 cfu/L).

As regards temperature, *Legionella* species demonstrate the ability to survive in a wide spectrum of temperatures, ranging from lower than 20°C, maintaining their ability to multiply in slow rates in some of these temperatures, to as high as 70°C, although for short periods of time, while displaying differences in their preferred temperature ranges for growth and survival depending on the species and the serogroup. *Legionella pneumophila* can be isolated from water temperatures ranging from 5.7 to 63°C, while proliferating at temperatures ranging between 25 and 45°C, peaking at 35 ± 2°C (Rasheduzzaman et al., 2020; Papagianeli et al., 2021). Even though the multiplication rate of *Legionella* increases in temperatures close to the optimal, the maintenance of a stable, albeit lower, population seems to be steadier in lower temperatures such as 25°C. This can be explained by the lower metabolic rates of the bacteria in these temperatures (Schwake et al., 2015). These low metabolic rates also seem to account for the low numbers of *Legionella* in biofilms forming in temperatures as low as 20°C, where the high number of *Acanthamoeba* theoretically provides a substrate for *Legionella* growth (Rogers et al., 1994).

As *Legionella* species exhibit limited survival in temperatures higher than 50°C, a constant high temperature can be used as a means of thermal disinfection. However, the current guidance for hot water temperature (50 to 55°C) seems inadequate as it is rejected by a number of studies which favor a range of temperatures from 55°C to 60°C (Rasheduzzaman et al., 2020; Papagianeli et al., 2021). While species such as *L. longbeachae*, a species found commonly in potting soil as well as in water systems are susceptible enough to a water temperature of 50°C, *L. pneumophila*, and especially the common serogroup 1, seems to be resistant to this temperature, whereas serogroup 7 seems to be resistant to temperatures as high as 55°C (Borella et al., 2005; Cervero-Arago et al., 2015). When it comes to temperatures higher than 55°C they were found to be significantly associated with decreased *Legionella* colonization coming in contrast with the positive correlation between water temperature <55°C and *Legionella* contamination (Rasheduzzaman et al., 2020). Above 60°C, all *L. pneumophila* serogroups exhibit similar behavior, surviving for several minutes (30 min 70°C). *Legionella* show higher tolerance for heat when associated with amoeba and biofilms, thus showing the need to examine other prevention factors such as disinfectants and nutrient availability (Cervero-Arago et al., 2015). Knezevic et al. recorded a statistically significant difference in the median temperature among the samples positive for Legionella and those negative for the bacterium, whether the water was warm or cold (Knezevic et al., 2022). The same study also concluded that *L. pneumophila* serogroup 1 showed a preference for higher temperatures compared to the rest serogroups.

Based on the results of our study, according to the generalized linear model both hot and cold-water supply seemed to affect the presence of *Legionella* when tested in separate at the cut-off proposed by the national legislation (1000 cfu/L). When applying a univariate approach, the exposure to temperature (under 25°C for cold water and over 47°C for hot water) also showed a high association with *Legionella*.

Moreover, according to the logistic regression and the binomial model created, exposure to different water temperatures seemed to be the covariate with the greater association with *Legionella*. In particular, cold water seemed to have positive linear relationship with *Legionella* while hot water seems to have negative linear relationship with the bacterium. Similar results were obtained when Count and Hurdle models were also applied.

In general, it seems that it is agreed that a hot water temperature of <50°C is a risk factor for *Legionella* contamination, but there still seems to exist a controversy when it comes to the temperature range between 50°C and 60°C. There are studies that have suggested that contamination at temperatures between 50°C and 60°C or >60°C don’t differ significantly and accept the guideline of >55°C (Volker and Kistemann, 2015), while others have pointed out the survival of a colonization of water distribution systems even at >60°C and hold temperatures as high as 57°C as a risk factor if water chlorination/hygiene is inadequate. Thus, these studies strictly suggest a temperature of over 60°C unless these other parameters can be regularly and closely monitored (Domenech-Sanchez et al., 2022).

Of interest, temperature also affects both the physico-chemical parameters which include chlorine decay and the absorption of chemicals and determine the formation of biofilms as well as the dynamics of formed biofilms allowing *Legionella* to detach and spread through the water system (Calero Preciado et al., 2021).

As regards disinfection, chlorination of hot water systems is one of the two major preventive measures against *Legionella* contamination, alongside thermal disinfection, and it can be used either as a secondary method of disinfection or as an installation disinfection method (hyper-chlorination). It is recommended by the WHO that drinking water has a minimum chlorination of 0.2 mg/L or 0.5 mg/L, depending on the risk of said water source for contamination (Cervero-Arago et al., 2015). The chlorine levels of the water seem to correlate negatively with *L. pneumophila* growth except for serogroup 1 which seems to be more resistant to the disinfectant. Knezevic et al. reported a simultaneous presence of chlorine along with Legionella, however the presence of the bacterium was reported at low to very low levels of the disinfectant (Knezevic et al., 2022).

According to our results and regardless of the models used, only total *Legionella*, dis-regarding the cut-off value (50 or 1000 cfu/L, revealed a statistically significant value and only when chlorine was treated as a qualitive parameter.

Other studies have also pointed out the negative correlation between chlorine concentration and *Legionella* growth however, they note that inside *Acanthamoeba* vesicles, where *Legionella* grow parasitically especially in biofilms, there is an increase in chlorine resistance. However, increasing chlorination is not a viable option as it would cause odor and taste problems to the water while also posing a health threat itself (Falkinham et al., 2015).

Apart from the physico-chemical factors, we also investigated the potential influence of other pathogenic microorganisms on the presence of *Legionella*. The pathogens whose presence and dynamics are currently studied to assess the water quality, mainly include Enterococci, *E. coli* and other coliforms. However, these pathogens’ presence or absence fails to determine the growth of opportunistic pathogens (OPs), which are usually not of Enteric or Fecal origin (Felfoldi et al., 2010; Mapili et al., 2020). It has also been suggested that the presence of certain other opportunistic pathogen species or other *Legionella* species in biofilms promote *Legionella* growth through increased resistance to temperature and disinfectants by horizontal gene transfer (Girolamini et al., 2022). This however is still controversial as studies suggest both an association and a lack thereof between the growth of *Legionella* and other OPs such as *P. aeruginosa* (Masaka et al., 2021; Atnafu et al., 2022).

According to the generalized linear model that we created, *E. coli*, coliforms and *P. aeruginosa* seemed to have a positive correlation with *L. pneumophila* sg 1, *L. pneumophila* and total *Legionella*. When testing our results by univariate analysis, *Pseudomonas* species and total coliforms showed a statistically significant impact on the presence of *Legionella* regardless of the species. It seems that increased numbers of *E. coli*, coliforms and *P. aeruginosa* may be present with increased numbers of *Legionella*. It is not clear whether the parallel presence of *E. coli* and coliforms is due to adequate presence of nutrients or the presence of biofilm that can provide a rescue room for these bacteria. To assess this issue the study of biofilm presence and perhaps of amoebae should have been necessary.

Our multivariate analysis showed that *P. aeruginosa* seems to prefer lower temperatures than *Legionella* for its survival and multiplication; a similar observation was recorded following PCA analysis. On the contrary, hot water exerts a negative effect on all pathogens tested when chlorine and temperature were not considered as variables. Free chlorine seems to exert a similar effect on all pathogens.

It has been suggested that *Legionella* could be used as an alternate indicator of water quality. On the other hand, the need for specific culture media, the different sensitivity to certain factors such as disinfectants between *Legionella* and other opportunistic pathogens and Fecal bacteria and the existence of viable but not culturable *Legionella* limit the pathogen’s potential usage as a sole indicator of water quality and it is suggested that it should be used in conjugation with other “typical” pathogens (Zhang et al., 2021b).

As far as HPC is concerned, it is considered an early indicator of microbial growth within a water distribution system since it signalizes the availability of organic nutrients, formation of biofilms and the presence of amoebae (Stojek and Dutkiewicz, 2011; Wingender and Flemming, 2011). HPC is used to monitor and assess the quality of water, as its increase is indicative of a post treatment biological activity in a hot water system, essentially signifying the existence of biofilms or the nutritional conditions needed for their formation (Bargellini et al., 2011; Volker et al., 2016). Formation of biofilms allows for *Legionella* to grow both intracellularly, as parasites to protozoan hosts, and extracellularly, being supported by various cyanobacterial species (Rogers et al., 1994). Specifically, *Legionella* grows intracellularly in vesicles contained in various *Acanthamoeba* spp. and extracellularly by harvesting carbon and energy sources excreted by other bacteria (Declerck et al., 2009; Stojek and Dutkiewicz, 2011). Biofilms also provide protection from disinfectants and temperature while serving as a substrate for exchange of genetic material and play a pivotal role in the maintenance of *Legionella* through the water system as parts of them get detached as a result of many factors such as temperature (Whiley et al., 2019; Calero Preciado et al., 2021; Girolamini et al., 2022).

While it stands to reason that *Legionella* occurrence could correlate with HPC, studies have shown both the existence and the lack between HPC and *Legionella* occurrence and it should be noted that the studies which do suggest a correlation point out that their results are limited by other factors such as temperature, with >50°C not showing any correlation between HPC and *Legionella* occurrence (Serrano-Suarez et al., 2013; Whiley et al., 2019). Some other studies have shown a significant correlation between *Legionella* contamination and HPC at specific temperatures, for example at 30°C, while others have only associated the two parameters in certain temperatures and only for very high HPC values (>100 CFU/ml) revealing the need for HPC and biofilm to be examined with more parameters such as water stagnation (Bargellini et al., 2011; Volker et al., 2016). A recent study (Knezevic et al., 2022) reported that HPC was positively associated with *Legionella* only when HPC was recorded following incubation on blood agar plates at 36°C for four days. At less days of incubation or when yeast extract was used as a medium, no significant correlation was reported. These studies perhaps indicate that HPC may not be a rigid parameter that can be used to monitor the presence of Legionella.

Stagnation of water occurs when there is an inadequate water flow rate, often due to a lack of a circulation pump or the existence of a physical dead leg in the hot water system. The stagnation zones allow bacteria to create biofilms providing both the time necessary for their formation and by making it difficult to maintain high temperatures and disinfectant concentrations (Volker et al., 2016). Although we did not study the role of water stagnation in our study, there have been studies that agree upon the correlation of water stagnation and *Legionella* contamination. Some authors attribute it to the dissipation of chlorine, going as far as to point out the correlation of winter and spring, the seasons with the lowest water usage and thus the highest water stagnation, with *Legionella* growth (Al-Bahry et al., 2011; Zhang et al., 2021b).

In our study we chose to test for the effect of number of stars and rooms upon the presence of *Legionella* in the water samples collected. The choice of these two factors was made as an indirect way to test for the effect of the complexity of a water distribution system assuming that the higher the number of stars and rooms the bigger the building and the more complex the water distribution system. However, we failed to show any correlation between the presence of *Legionella* compared to the number of stars and rooms of the hotel tested irrespective of the model, or the analysis chosen.

Other studies have revealed that the age and height of a building are positively correlated with *Legionella* contamination of the water distribution system, possibly because they make it difficult for proper water temperatures to be maintained throughout the entirety of its extent, especially at higher floors. An age of 20 or more years and a number of floors over 10 are also considered risk factors (Masaka et al., 2021; Atnafu et al., 2022; Girolamini et al., 2022). On the contrary, a newer study failed to exhibit a correlation with *Legionella* growth with the age of a water distribution system pipes (Wingender and Flemming, 2004). In general, however, it seems that lower floors of taller buildings are not protected, as *Legionella* spreads to them from the higher floors (Borella et al., 2005; Gamage et al., 2022). Furthermore, the outlet distance has also been associated with *Legionella* contamination, as the outlets further away from temperature decrease, biofilm formation and stagnation of water (Volker et al., 2016).

According to the analysis that we performed, the proximity of the sample point to the boiler seemed to exert an effect (positive or negative based on the distance from the boiler) to *L. pneumophila* sg 1 but not total *Legionella* regardless of the cut-off used.

Although we did not test for the effect of nutrients, *Legionella* species have very low requirements to survive and grow. The presence of copper seems to limit the bacterium’s growth. The hardness and the pH of the water seem to influence *Legionella*’s growth, both directly and indirectly, as a low pH leads to corrosion of pipe walls which allows for biofilms to develop (Borella et al., 2005; Volker et al., 2016).

Other studies agree that the release of nutrients in the water distribution system, possibly by like rubber coated valves favor the formation of biofilms and some of these nutrients such as ammonia have been correlated with *Legionella* contamination (Wingender and Flemming, 2004; Zhang et al., 2021b).

We also tried to test for the effect of direct or indirect sampling. The results that we revealed were ambiguous since no relationship was calculated at a cut-off of 1000 cfu/L for *Legionella* and of 50 cfu/L for *L. pneumophila* sg 1 while a negative effect was calculated at a cut-off of 50 cfu/L for *Legionella* and a positive effect at a cut-off of 1000 cfu/L. Certainly, more studies need to be carried out to study the potential effect of flushing on the detection of *Legionella*.

Lastly, we did not reveal any correlation between the presence of *Legionella* compared to the number of hotels situated at the region of sampling when applying complicated analysis, while when we focused strictly on the number of hotels tested in the area from which the samples were collected, a statistically significant p-value was calculated regardless the type of analysis followed. Again, more studies need to be carried out to reveal any potential effects lying behind.

The findings of the current study further support the belief that simple recording of temperature, chlorine, Legionella concentrations, etc. is no longer enough to minimize the risk of colonization of water distribution systems. A more holistic approach is need to properly survey Legionella presence since the latter seems to be affected by far more factors than just temperature and chlorine (or any other disinfectant). There is a need to take under consideration and routinely test for the presence of opportunistic and other bacteria since their presence may either act as indicator of Legionella presence or may enhance the presence of the bacterium. Other epidemiological factors should also be considered when trying to set up a guidance for the risk factors that affect Legionella. Water stagnation and age/type of pipelines should be included into the risk factors considered. There have been several cases of hotel units that try to get rid or even minimize the presence of the bacterium in their water distribution systems without any success. Simple hyper-chlorination and/or heat shock do not always prove enough to minimize the risk. To keep the system under control and to maintain an equilibrium of the bacterium concentration in the water distribution system, there is a need to remove all these factors that enhance the survival and proliferation of Legionella.

The results of this study add up to the existing knowledge recorded from past studies on the area and could be used as a guide to proper hotel management in implementing effective water treatment strategies to reduce Legionella risk in water distribution systems.

## Limitations of the study

Certainly, the results of the current study may be limited by the specific geographic focus on Crete; further studies in diverse locations may provide more generalized insights on the effect of the above-mentioned factors on Legionella. Moreover, although we tried to implement as many statistical approaches as possible and study as many parameters as possible, it would be inevitable to include all of them. Under this term, we need to point out that part of the study was performed during the SARS-CoV-2 pandemic where several hotels either remained closed or started their operation with great delay during the summers. These prolonged closures may have affected the microbiological quality of the water distribution systems and may have made it more difficult to clean the pipelines from any microorganisms, biofilms, etc. Therefore, if the same study was carried out before the pandemic different results could have been recorded. Moreover, we did not test for the potential effect of stagnation of water or the protective effect that amoebae may confer to *Legionella*. We did not also study the presence of viable but not culturable forms of *Legionella* or the potential effect that these forms of the bacteria may have had upon our results. Although we tried to investigate the effect of several epidemiological factors associated with the hotel (for example the number of rooms or stars), we did not study the effect of seasonality upon our results. We did not also test for the presence of chemical agents, such as Mg, Mn, Fe, etc., that may affect the presence of Legionella in the water distribution systems. Of course, in case we needed to do so, we would have to repeat all our analyses for each season separately. Application of different models did not always give us back similar results. Under this concept, processing of our data with neural networking or artificial intelligence may had provided us with different outcomes. In general, more studies could or should focus on these aspects as well.

## Conclusions

Plumbing drinking water systems show great short- and long-term variability in terms of the presence and concentration of *Legionella*. Regular testing by classical culture methods cannot always reliably reveal contamination risks. There is a need for a longitudinal sampling approach, better risk assessment and better specification of the number of samples that need to be collected to be representative of the water distribution system. We need to consider the entire drinking water plumbing system as an ecological system and each outlet as an ecological niche. The analysis of single parameters specific to individual sampling points cannot reliably predict the possibility of *Legionella* contamination. The application of empirical modeling using logistic regression can provide a valuable contribution to a better risk assessment for hydraulic drinking water systems than conventional culture-based detection methods. Moreover, the use of different models may provide different results. It is crucial to assess all these parameters prior to sampling since this can help: in risk assessment, in the prioritization of risk areas, in identifying suitable sampling points, in determining control measures within a building and in the development of a water safety plan for a specific building.

The results of the current study provide further knowledge on the study of *Legionella* presence in water distribution systems. Several factors may affect its presence and multiplication and certainly more complex approaches are needed to control the bacterium.

## Data availability statement

The raw data supporting the conclusions of this article will be made available by the authors, without undue reservation.

## Author contributions

DC designed the experiment, did the laboratory testing and wrote the manuscript. VS wrote the manuscript and did the laboratory testing. AN wrote the manuscript. M-OD did the statistical analysis. TL did the statistical analysis. NT did the laboratory testing. RM did the laboratory testing. CP collected samples. AP did the supervision. All authors contributed to the article and approved the submitted version.
